# Health‐related quality of life and survival outcomes for patients with major depressive disorder and anxiety: A longitudinal study in pancreatic ductal adenocarcinoma

**DOI:** 10.1002/cam4.6577

**Published:** 2023-09-25

**Authors:** Yuchen Ji, Haoda Chen, Yuxuan Yang, Yiran Zhou, Hongzhe Li, Haiyan Jin, Junjie Xie, Baiyong Shen

**Affiliations:** ^1^ Department of General Surgery, Pancreatic Disease Center, Ruijin Hospital Shanghai Jiao Tong University School of Medicine Shanghai China; ^2^ Institute of Translational Medicine, Shanghai Jiao Tong University Shanghai China; ^3^ Research Institute of Pancreatic Disease Shanghai Jiao Tong University School of Medicine Shanghai China; ^4^ State Key Laboratory of Oncogenes and Related Genes Shanghai China; ^5^ Department of Psychiatry Ruijin Hospital, Shanghai Jiao Tong University School of Medicine Shanghai China

**Keywords:** 1‐year survival outcomes, anxiety, chemotherapy compliances, major depressive disorder, pancreatic ductal adenocarcinoma, quality of life

## Abstract

**Background:**

Major depressive disorder (MDD) and anxiety were recognized in treating pancreatic ductal adenocarcinoma (PDAC). This longitudinal study identified risk factors for MDD and anxiety and established associations with patients' quality of life (QoL) and survival outcomes.

**Materials and Methods:**

We used PHQ‐9 and GAD‐7 questionnaires to diagnose MDD and anxiety in PDAC patients between October 2021 and March 2022 at a Chinese center. Characteristics and clinical data were analyzed for risk factors and EORTC QLQ‐C30 questionnaire was administered for QoL before the first chemotherapy. Furthermore, chemotherapy compliance and 1‐year survival were compared during follow‐up.

**Results:**

MDD and anxiety occurred in 51.8% and 44.7% of 114 patients over the half‐year period. Employment at work (odds ratio [OR]: 5.514, *p* = 0.001; OR: 3.420, *p* = 0.011) was an independent risk factor, while radical surgery (OR: 0.342, *p* = 0.034; OR: 0.238, *p* = 0.004) was a protective factor. Several aspects of decreased QoL were discovered after their onsets. Higher incidences of physical disorders (*p* = 0.004; *p* < 0.001), mental disorders (*p* = 0.001; *p* < 0.001), anti‐therapy emotions (*p* = 0.002; 0.001), and chemotherapy suspensions (*p* = 0.001; *p* = 0.043) were observed. Furthermore, the 1‐year mortalities for all patients and those receiving radical surgeries were correlated with MDD (*p* = 0.007; 0.036) and anxiety (*p* = 0.010; 0.031).

**Conclusions:**

MDD and anxiety are common in PDAC patients and correlated with poor QoL and survivals. Therefore, appropriate mental management is required in future.

## 1 INTRODUCTION

Previous epidemiological data have shown that the incidence of major depressive disorder (MDD) in cancer patients can be four to five times much higher than in the general population.^1^, ^2^ Anxiety and MDD are frequently associated and have been proven to increase mortality in various malignancies significantly.^3^ Among digestive system malignancies, patients with MDD and anxiety have been observed to have higher incidences of postoperative complications, longer hospital stays, and worse prognoses.^3^, ^4^

Pancreatic ductal adenocarcinoma (PDAC) is a highly lethal tumor with a 5‐year survival rate of only 7.2%, ranking it sixth among China's leading causes of malignancy‐related deaths.^5^ Previous research has shown that the risk of MDD or anxiety can be especially high in patients with PDAC.^3^, ^6^, ^7^ For instance, the overall prevalence of MDD in patients with PDAC ranges from 12% to 78%, with differences in study design, assessment methods, and disease stages.^8^

Progression and prognosis can be correlated with mental disorders in patients with both resectable and advanced tumors receiving palliative care only. Patients with mental disorders were found to be more likely to engage in high‐risk behaviors and receive less social care. They were also less inclined to seek proper medical care and follow clinicians' guidelines.^9^, ^10^, ^11^ Therefore, MDD and anxiety can significantly worsen the prognosis of malignant tumors and increase overall mortality by decreasing treatment compliance. However, symptoms caused by mental disorders, such as pain, fatigue, and weight loss, can mimic those caused by PDAC, leading to delayed identification of tumor progression.^10^, ^12^

In addition to prognosis, the health‐related quality of life (HR‐QoL) has received increasing attention in recent decades, especially in patients with severely advanced tumors. Evidence also shows that disease progression is associated with HR‐QoL deterioration in several tumor types.^13^ However, the impact of mental disorders on the quality of life (QoL) for PDAC patients remains understudied in the Chinese population.

We conducted a real‐world study at a high‐volume center in China to establish the association between the occurrence of MDD and anxiety with PDAC patients' QoL and survival outcomes as awareness of the role of mental disorders in cancer treatment rapidly develops. Furthermore, risk factors for MDD and anxiety development were investigated based on patient characteristics and clinical data.

## 2 MATERIALS AND METHODS

### 2.1 Patients' selection

We selected patients aged >18 years who received treatment for PDAC at Ruijin Hospital, affiliated with Shanghai Jiao Tong University School of Medicine, between October 2021 and March 2022 for MDD or anxiety assessments. The same group of surgeons treated all patients and they voluntarily signed informed consent forms for mental assessments and data collection. All patients were fully informed and cognitive of their state of illness and malignant property of PDAC. The exclusion criteria were as follows:(1) patients previously diagnosed with pancreatic neoplasms and received any treatment, (2) those who refused to participate in the questionnaire survey during further follow‐up after discharge, (3) patients without important clinical data recorded, (4) patients with a previous medical history of mental disorders, and (5) patients that experienced severe complications whose Clavien–Dindo classifications^14^ were IV–V. All work in this study was conducted according to the provisions of the Declaration of Helsinki (revised in 2013).

### 2.2 Hospital stay treatments and data collections

All patients diagnosed with PDAC underwent curative surgery if the tumors could be radically resected. Other patients whose tumors could not be radically removed underwent histopathological biopsy through laparoscopic exploration. All patients received their first chemotherapy nearly 1 month after discharge.

We collected the patients' clinical data at the same time of the metal assessments for MDD and anxiety, including age, sex, American Society of Anesthesiologists (ASA) class, body mass index (BMI), hospital stay, symptoms, comorbidities, and habits (smoking and alcohol use). The laboratory data included hemoglobin, serum total bilirubin, albumin, and CA199 levels. Treatments (palliative or radical) and tumor stages were also recorded. Furthermore, we collected patients' non‐biological factors, such as medical insurance, income, education level, marital status, working status, and self‐care ability, as they were proven to affect survival outcomes in our previous study on PDAC patients.^15^

### 2.3 Questionnaire of mental assessments and quality of life

The MDD and anxiety in our study were designed to be cancer‐related and treatment‐related, therefore the first questionnaire was conducted for mental assessments approximately 2–3 weeks after discharge from hospital. The Patient Health Questionnaire‐9 (PHQ‐9)^16^ and Generalized Anxiety Disorder‐7 (GAD‐7)^17^ questionnaires were used to easily diagnose MDD or anxiety and grade their severity in patients with PDAC. A PHQ‐9 total score ≥5 indicated MDD, while a GAD‐7 total score ≥5 indicated anxiety. The severity of MDD was graded as Grade I (5–9), Grade II (10–14), Grade III (15–19), and Grade IV (20–27). Anxiety severity was graded as Grade I (5–9), Grade II (10–14), and Grade III (15–21).

The second questionnaire survey was conducted prospectively just before the first beginning of the chemotherapy session to assess the QoL of patients with PDAC. The European Organization for Research and Treatment of Cancer Core (EORTC QLQ‐C30)^18^ was used to evaluate several aspects of the QoL of patients with PDAC, including five functional scales (physical, role, emotional, cognitive, and social functioning), three symptom scales (fatigue, nausea and vomiting, and pain), six single‐item scales (dyspnea, insomnia, appetite loss, constipation, diarrhea, and financial difficulties), and a global health scale. After linear transformation, higher scores on the functional and global health scales indicated a better QoL. In contrast, higher scores on the symptom and single‐item scales indicated a worse QoL.

### 2.4 Chemotherapy compliance and follow‐up

After treatment for PDAC, all patients were followed‐up at regular intervals via telephone for routine clinical care every 3 months during the first year in our center. Follow‐up ended when the patient died or contact was lost. Chemotherapy compliance was also prospectively monitored in this study by querying about the patients' physical status (e.g., pain, dyspnea), mental status (e.g., aggressive, depressive), anti‐therapy emotions, and therapy suspensions. The anti‐therapy emotions were mainly judged by patients' or their families' subjective descriptions, such as partial rejection but final continued medication after persuasion or complete rejection against the chemotherapy. Our most recent follow‐up was conducted in March 2023, and patient survival data were censored. Every patient in our study received at least 1 year of follow‐up after discharge from the hospital. We focused on patients' 1‐year overall survival (OS) results, which were defined as the time from treatment to death due to any cause. We also collected recurrence or metastasis data for patients who underwent radical surgery and focused on the 1‐year disease‐free survival (DFS) results.

### 2.5 Statistical analysis

All statistical results were analyzed using the SPSS software, and statistical significance was set at *p* < 0.05. The correlation of patients' PHQ‐9 and GAD‐7 scores was assessed through Pearson method. Categorical variables are presented as numbers (percentages) and were analyzed using either chi‐squared or Fisher's exact tests. Student's *t*‐test was used to compare normally distributed continuous variables, presented as means ± standard deviation. The results of non‐normally distributed continuous variables are presented as medians (interquartile ranges) and compared using the Mann–Whitney *U*‐test. Potential risk factors for MDD and anxiety were first analyzed, including demographics, non‐biological factors, symptoms, comorbidities, habits, laboratory tests, and tumor characteristics. Factors with statistical significance were then enrolled into a multivariate logistic regression model to identify the independent factors.

Additionally, MDD, anxiety, chemotherapy compliance, and 1‐year DFS were included in the univariate analysis of factors influencing 1‐year survival results. Odds ratios (OR) and 95% confidence interval (CIs) were determined. Furthermore, boxplot diagrams were utilized to provide more intuitive presentations of the patients' QoL, stratified by MDD and anxiety status.

## 3 RESULTS

### 3.1 Study population

**FIGURE 1:**
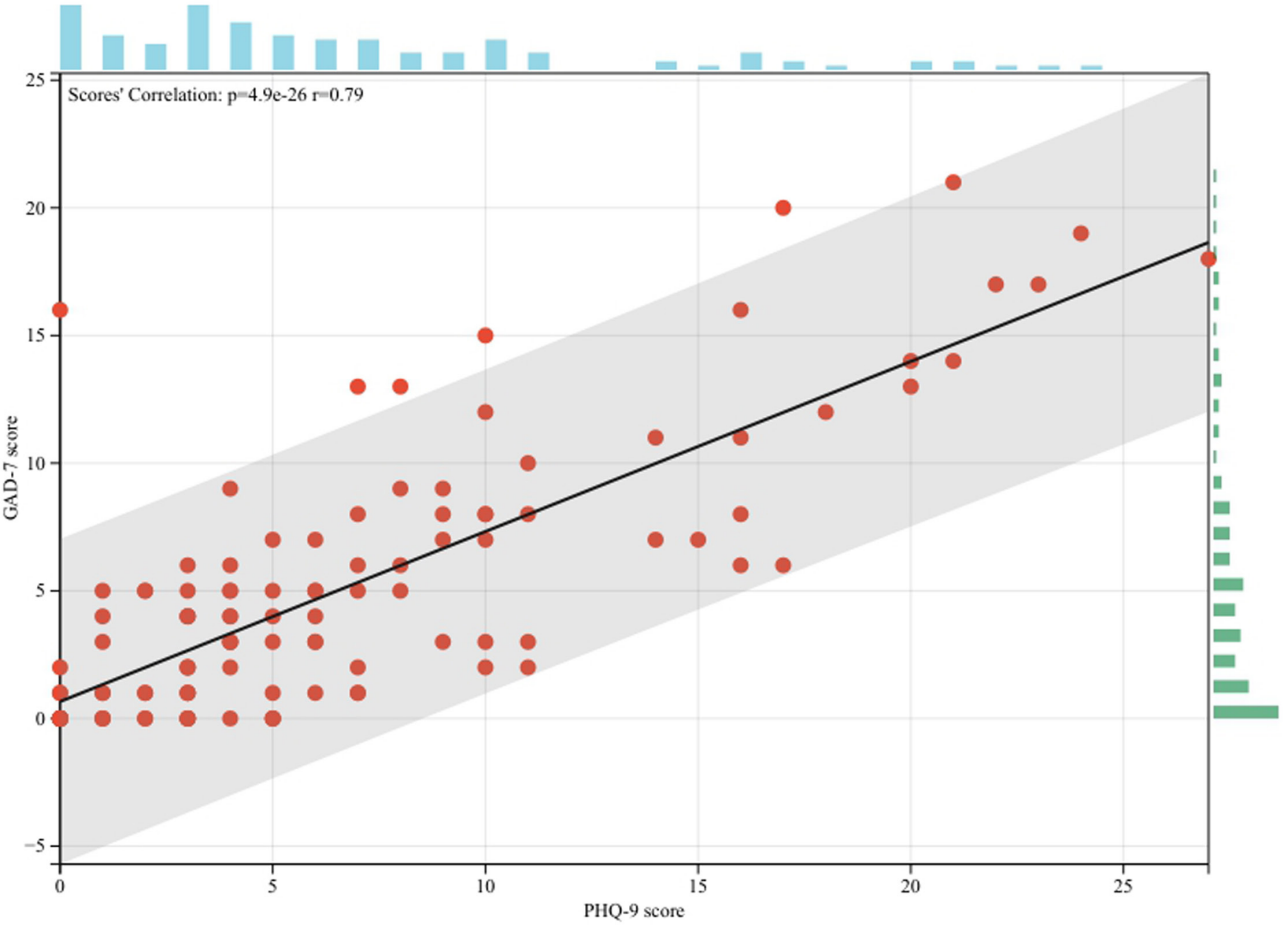
Correlation and distribution of 114 patients' PHQ‐9 and GAD‐7 scores. The two scores were linearly correlated (*p* < 0.001), with a coefficient of 0.79. PHQ‐9, Patient Health Questionnaire‐9; GAD‐7, Generalized Anxiety Disorder‐7.

During the study period, 325 PDAC patients were eligible according to our selecting criteria, while only 114 patients (35.1%) consented to be enrolled in this longitudinal study and further questionnaire surveys. All of these 114 patients were not lost in our regular follow‐up. Among them, 59 (51.8%) developed MDD, and 51 (44.7%) developed anxiety. Thirty patients had Grade I MDD, while 13, 8, and 8 had Grades II, III, and IV, respectively. Regarding anxiety, 32 patients had Grade I, while 10 and 9 had Grades II and III, respectively. Figure 1 shows the correlation between all patients' PHQ‐9 and GAD‐7 scores; the results were statistically significant (*p* < 0.001). The two scores had a positive linear correlation, with a correlation coefficient of 0.79, and 41 (36.0%) patients developed both MDD and anxiety after PDAC confirmation.

Patients with MDD were less insured in the entire cohort except for the national medical insurance (59.3% vs. 80.0%; *p* = 0.029). They were also more likely to be employed (40.7% vs. 10.9%; *p* = 0.001) than those without MDD. Furthermore, patients whose tumors were not radically resected and those who only received palliative treatment were more likely to develop MDD (32.2% vs. 14.5%; *p* = 0.046). Similar results were observed in the analysis of anxiety, with the factors mentioned above being significantly different (Table 1).

**TABLE 1:** Patients' characteristics depending on major depressive disorder or anxiety status.

Variables	Without MDD (*n* = 55)	With MDD (*n* = 59)	*p*‐value	Without anxiety (*n* = 63)	With anxiety (*n* = 51)	*p*‐value
Age (mean [SD]), years	59.09 (9.56)	62.02 (9.69)	0.108	60.21 (9.52)	61.10 (9.98)	0.628
Sex, *n* (%), male	27 (49.1)	38 (64.4)	0.144	33 (52.4)	32 (62.7)	0.357
ASA class, *n* (%)						
I	26 (47.3)	27 (45.8)	1.000	29 (46.0)	24 (47.1)	1.000
II‐III	29 (52.7)	32 (54.2)		34 (54.0)	27 (52.9)	
BMI (mean [SD]), kg/m^2^	23.14 (3.69)	23.03 (3.94)	0.877	23.14 (3.49)	23.00 (4.19)	0.841
*Nonbiological factors*						
With additional medical insurance, *n* (%)	44 (80.0)	35 (59.3)	0.029	50 (79.4)	29 (56.9)	0.017
Income, *n* (%)						
<￥2000/month	11 (20.0)	8 (13.6)	0.572	11 (17.5)	8 (15.7)	0.966
￥2000–5000/month	15 (27.3)	20 (33.9)		19 (30.2)	16 (31.4)	
>￥5000/month	29 (52.7)	31 (52.5)		33 (52.4)	27 (52.9)	
With college education, *n* (%)	9 (16.4)	10 (16.9)	1.000	11 (17.5)	8 (15.7)	1.000
Married, *n* (%)	55 (100.0)	58 (98.3)	1.000	62 (98.4)	51 (100.0)	1.000
Working status, *n* (%)						
Retire or unemployed	49 (89.1)	35 (59.3)	0.001	53 (84.1)	31 (60.8)	0.009
Employed	6 (10.9)	24 (40.7)		10 (15.9)	20 (39.2)	
Self‐care ability, *n* (%), absent	5 (9.1)	4 (6.8)	0.913	4 (6.3)	5 (9.8)	0.741
*Symptoms*						
Tumor pain, *n* (%)	27 (49.1)	26 (44.1)	0.591	30 (47.6)	23 (45.1)	0.788
Weight loss >3 kg, *n* (%)	13 (23.6)	19 (32.2)	0.419	14 (22.2)	18 (35.3)	0.182
*Comorbidity*						
Diabetes mellitus, *n* (%)	16 (29.1)	16 (27.1)	0.980	17 (27.0)	15 (29.4)	0.938
Hypertension, *n* (%)	22 (40.0)	21 (35.6)	0.770	27 (42.9)	16 (31.4)	0.287
Cardiac or cerebral diseases, *n* (%)	8 (14.5)	9 (15.3)	1.000	8 (12.7)	9 (17.6)	0.636
*Habits*						
Smoke, *n* (%)	11 (20.0)	11 (18.6)	1.000	11 (17.5)	11 (21.6)	0.754
Alcohol, *n* (%)	12 (21.8)	12 (20.3)	1.000	12 (19.0)	12 (23.5)	0.724
*Laboratory tests*						
Hb (median [IQR]), g/L	132 (118–140)	129 (115–139)	0.755	127 (117–138)	130 (115–142)	0.536
TBil (median [IQR]), μmol/L	15.5 (10.5–24.1)	15.6 (11.4–30.0)	0.466	17.8 (11.8–40.8)	13.6 (10.5–23.3)	0.064
ALB (median [IQR]), g/L	41 (38–44)	39 (35–43)	0.092	40 (37–44)	41 (36–44)	0.934
CA199 > 37 U/mL, *n* (%)	35 (63.6)	44 (74.6)	0.288	44 (69.8)	35 (68.6)	1.000
*Tumor characteristics*						
Treatments, *n* (%)						
Palliative treatments	8 (14.5)	19 (32.2)	0.046	8 (12.7)	19 (37.3)	0.004
Radical surgery	47 (85.5)	40 (67.8)		55 (87.3)	32 (62.7)	
T stage ≥ III, *n* (%)	35 (63.6)	46 (78.0)	0.139	42 (66.7)	39 (76.5)	0.347
Metastasis, *n* (%)	5 (9.1)	13 (22.0)	0.102	6 (9.5)	12 (23.5)	0.075
N stage, *n* (%)						
N_x_ or N_0_	37 (67.3)	41 (69.5)	0.958	39 (61.9)	39 (76.5)	0.144
N_1_ or N_2_	18 (32.7)	18 (30.5)		24 (38.1)	12 (23.5)	
Hospital stay (median [IQR]), days	20 (15–27)	19 (15–29)	1.000	23 (17–30)	18 (14–26)	0.056

Abbreviations: ALB, albumin; ASA, American Society of Anesthesiologists; BMI, body mass index; CA199, carbohydrate antigen199; Hb, hemoglobin; IQR, interquartile range; MDD, major depressive disorder; SD, standard deviation; TBil, total bilirubin.

### 3.2 Independent risk factors for MDD and anxiety

All potential risk factors (*p* < 0.05) identified in the univariate analysis (Table 1), including additional medical insurance, working status, and treatments, were included in the multivariate logistic regression model (Table 2). Patients still employed at work (OR: 5.514, 95% CI: 1.965–15.475, *p* = 0.001; OR: 3.420, 95% CI: 1.332–8.784, *p* = 0.011) was an independent risk factor for MDD and anxiety development, while patients whose tumors could be radically resected was a protective factor (OR: 0.342, 95% CI: 0.127–0.924, *p* = 0.034; OR: 0.238, 95% CI: 0.088–0.640, *p* = 0.004). Patients without additional medical insurance (*p* = 0.137; 0.091) did not have a higher risk of developing MDD or anxiety.

**TABLE 2:** Multivariate analyses of risk factors for major depressive disorder and anxiety.

	MDD		Anxiety	
Risk factors	OR (95% CI)	*p*‐value	OR (95% CI)	*p*‐value
Additional medical insurance				
Without	Ref		Ref	
With	0.501 (0.201–1.247)	0.137	0.463 (0.190–1.130)	0.091
Working status				
Retire or unemployed	Ref		Ref	
Employed	5.514 (1.965–15.475)	0.001	3.420 (1.332–8.784)	0.011
Treatments				
Palliative treatments	Ref		Ref	
Radical surgery	0.342 (0.127–0.924)	0.034	0.238 (0.088–0.640)	0.004

Abbreviations: CI, confidence interval; MDD, major depressive disorder; OR, odds ratio.

### 3.3 Quality of life for patients with MDD and anxiety

**FIGURE 2:**
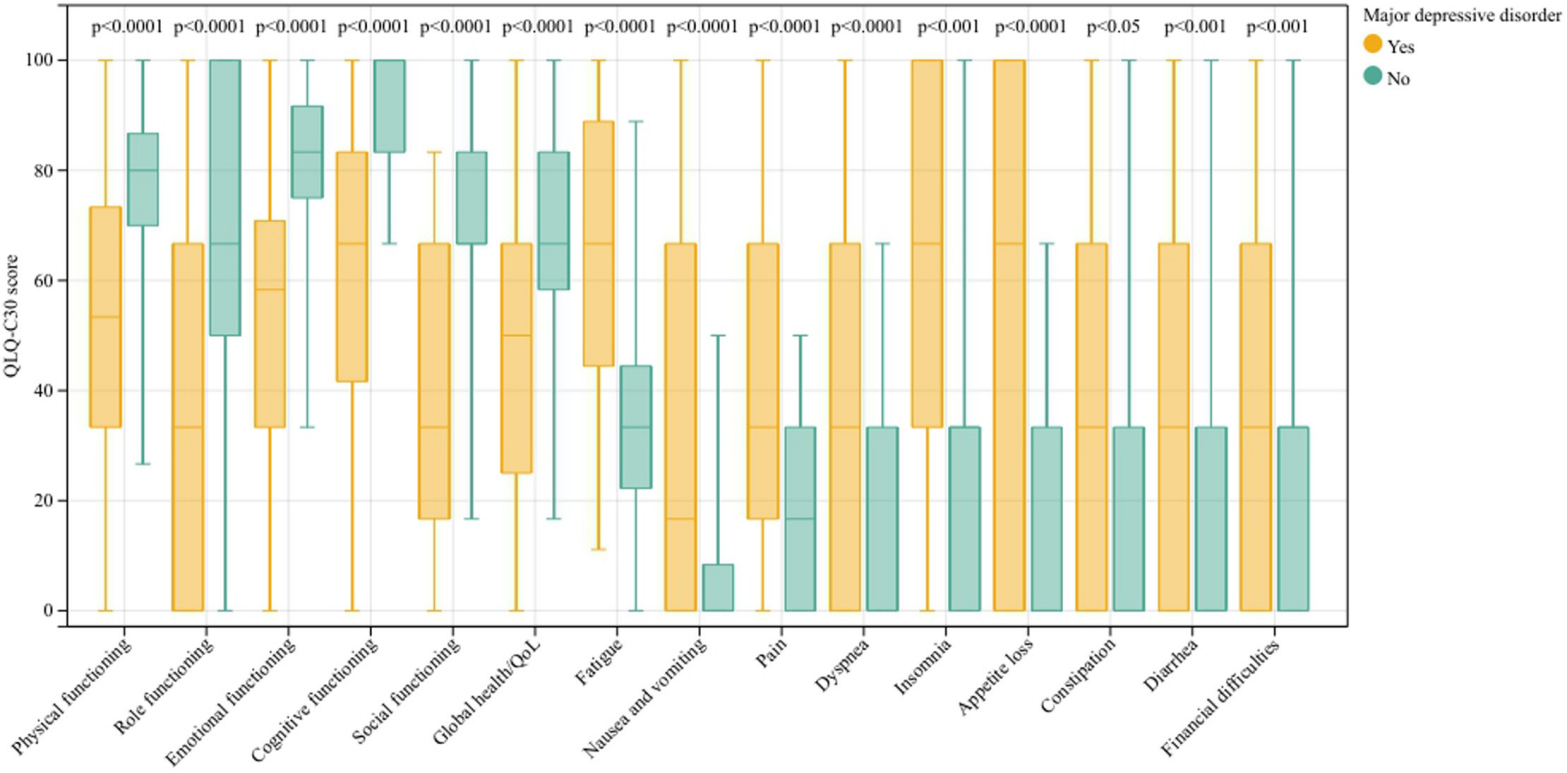
Patients' quality of life stratified by major depressive disorder status.

**FIGURE 3:**
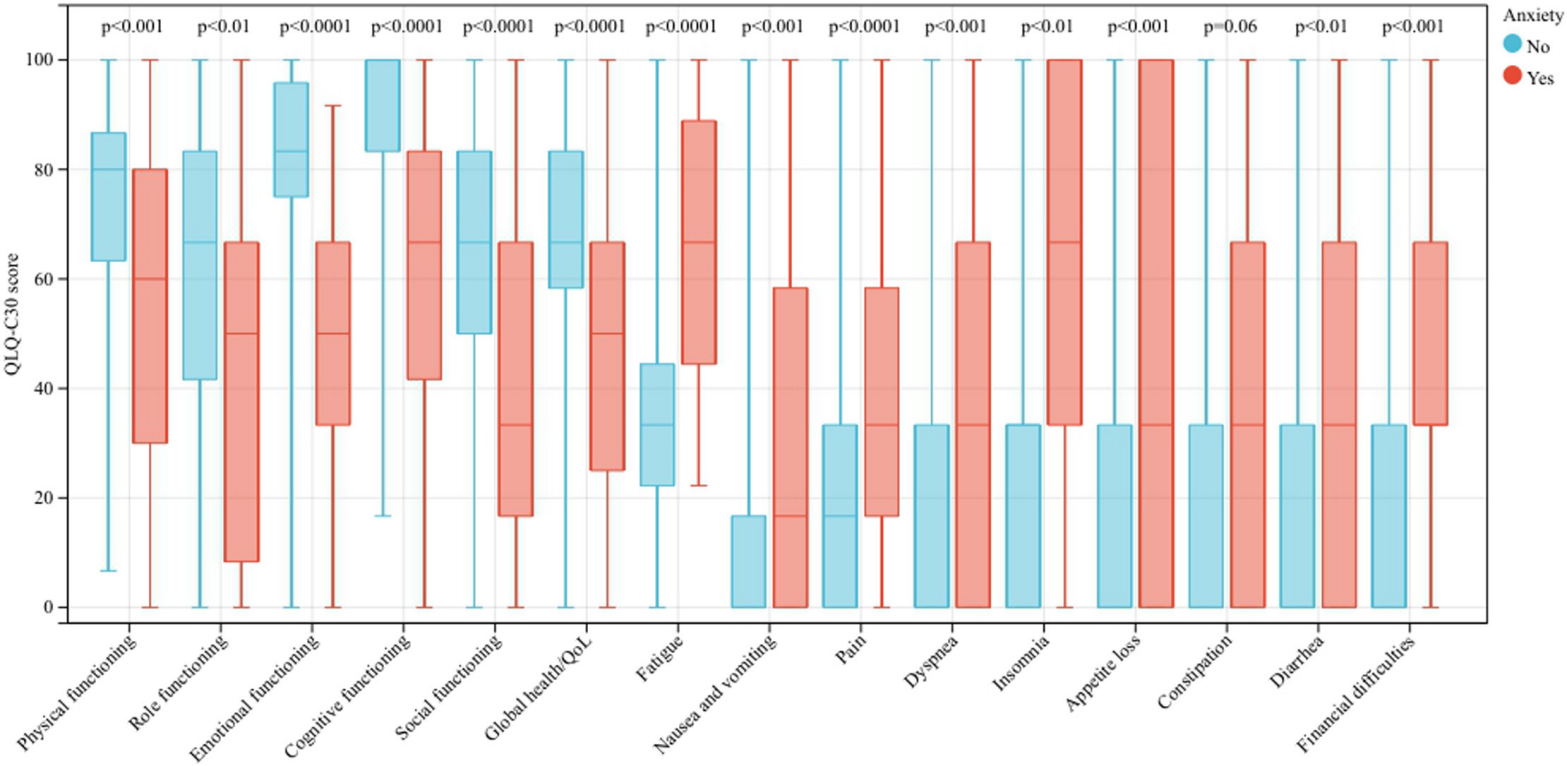
Patients' quality of life stratified by anxiety status.

Among patients diagnosed with MDD, physical functioning (53.3 vs. 80.0; *p* < 0.001), role functioning (33.3 vs. 66.7; *p* < 0.001), emotional functioning (58.3 vs. 83.3; *p* < 0.001), cognitive functioning (66.7 vs. 100.0; *p* < 0.001), social functioning (33.3 vs. 66.7; *p* < 0.001) and global health (50.0 vs. 66.7; *p* < 0.001) all presented lower scores indicating worse functions and life quality than patients without MDD. Furthermore, symptoms of fatigue (66.7 vs. 33.3; *p* < 0.001), nausea and vomiting (16.7 vs. 0.0; *p* < 0.001), and pain (33.3 vs. 16.7; *p* < 0.001), and single items including dyspnea (33.3 vs. 0.0; *p* < 0.001), insomnia (66.7 vs. 33.3; *p* < 0.001), appetite loss (66.7 vs. 0.0; *p* < 0.001), constipation (33.3 vs. 0.0; *p* = 0.028), diarrhea (33.3 vs. 0.0; *p* = 0.001), and financial difficulties (33.3 vs. 33.3; *p* = 0.001) also indicated a worse life quality status for MDD patients (Table 3; Figure 2). Similar results were observed when anxiety occurred, with statistical differences in all aspects except for constipation (*p* = 0.056) (Table 3; Figure 3).

**TABLE 3:** The life quality comparison of patients stratified by the status of major depressive disorder or anxiety.

Variables	Without MDD (*n* = 55)	With MDD (*n* = 59)	*p*‐value	Without anxiety (*n* = 63)	With anxiety (*n* = 51)	*p*‐value
Physical functioning	80.0 (66.7, 86.7)	53.3 (33.3, 73.3)	<0.001	80.0 (60.0, 86.7)	60.0 (26.7, 80.0)	<0.001
Role functioning	66.7 (50.0, 100.0)	33.3 (0.0, 66.7)	<0.001	66.7 (33.3, 83.3)	50.0 (0.0, 66.7)	0.007
Emotional functioning	83.3 (75.0, 91.7)	58.3 (33.3, 75.0)	<0.001	83.3 (75.0, 100.0)	50.0 (33.3, 66.7)	<0.001
Cognitive functioning	100.0 (83.3, 100.0)	66.7 (33.3, 83.3)	<0.001	100.0 (83.3, 100.0)	66.7 (33.3, 83.3)	<0.001
Social functioning	66.7 (66.7, 83.3)	33.3 (16.7, 66.7)	<0.001	66.7 (50.0, 83.3)	33.3 (16.7, 66.7)	<0.001
Global health/QoL	66.7 (58.3, 83.3)	50.0 (25.0, 66.7)	<0.001	66.7 (58.3, 83.3)	50.0 (25.0, 66.7)	<0.001
Fatigue	33.3 (22.2, 44.4)	66.7 (44.4, 88.9)	<0.001	33.3 (22.2, 44.4)	66.7 (44.4, 88.9)	<0.001
Nausea and vomiting	0.0 (0.0, 16.7)	16.7 (0.0, 66.7)	<0.001	0.0 (0.0, 16.7)	16.7 (0.0, 66.7)	0.001
Pain	16.7 (0.0, 33.3)	33.3 (16.7, 66.7)	<0.001	16.7 (0.0, 33.3)	33.3 (16.7, 66.7)	<0.001
Dyspnea	0.0 (0.0, 33.3)	33.3 (0.0, 66.7)	<0.001	0.0 (0.0, 33.3)	33.3 (0, 66.7)	<0.001
Insomnia	33.3 (0.0, 33.3)	66.7 (33.3, 100.0)	<0.001	33.3 (0.0, 33.3)	66.7 (33.3, 100.0)	0.002
Appetite loss	0.0 (0.0, 33.3)	66.7 (0.0, 100.0)	<0.001	0.0 (0.0, 33.3)	33.3 (0.0, 100.0)	<0.001
Constipation	0.0 (0.0, 33.3)	33.3 (0.0, 66.7)	0.028	0.0 (0.0, 33.3)	33.3 (0.0, 66.7)	0.056
Diarrhea	0.0 (0.0, 33.3)	33.3 (0.0, 66.7)	0.001	0.0 (0.0, 33.3)	33.3 (0.0, 66.7)	0.003
Financial difficulties	33.3 (0.0, 33.3)	33.3 (0.0, 66.7)	0.001	0.0 (0.0, 33.3)	33.3 (33.3, 66.7)	<0.001

Abbreviations: MDD, major depressive disorder; QoL, quality of life.

### 3.4 Chemotherapy compliances

All patients received chemotherapy, and patients with MDD or anxiety had higher rates of physical disorders (83.1% vs. 56.4%, *p* = 0.004; 88.2% vs. 55.6%, *p* < 0.001), mental disorders (71.2% vs. 38.2%, *p* = 0.001; 74.5% vs. 39.7%, *p* < 0.001), and anti‐therapy emotions (55.9% vs. 25.5%, *p* = 0.002; 58.8% vs. 27.0%, *p* = 0.001). Furthermore, during the chemotherapy period, patients with MDD (50.8% vs. 20.0%, *p* = 0.001) and anxiety (47.1% vs. 27.0%, *p* = 0.043) were more likely to suspend or terminate therapy for any cause (Table 4).

**TABLE 4:** Chemotherapy compliance of patients with major depressive disorder or anxiety.

Variables	Without MDD (*n* = 55)	With MDD (*n* = 59)	*p*‐value	Without anxiety (*n* = 63)	With anxiety (*n* = 51)	*p*‐value
Physical disorder during chemotherapy, *n* (%)	31 (56.4)	49 (83.1)	0.004	35 (55.6)	45 (88.2)	<0.001
Mental disorder during chemotherapy, *n* (%)	21 (38.2)	42 (71.2)	0.001	25 (39.7)	38 (74.5)	<0.001
Anti‐therapy emotions, *n* (%)	14 (25.5)	33 (55.9)	0.002	17 (27.0)	30 (58.8)	0.001
Chemotherapy suspension, *n* (%)	11 (20.0)	30 (50.8)	0.001	17 (27.0)	24 (47.1)	0.043

Abbreviation: MDD, major depressive disorder.

### 3.5 Influencing factors for 1‐year survival results

No patients were lost to follow‐up, and within 1 year of diagnosis and treatment for PDAC, 23 (20.2%) patients died, 18 of whom had MDD, and 16 had anxiety. Among the 87 patients who received radical surgeries in our cohort, the 1‐year recurrence or metastasis rates showed no significant differences when MDD or anxiety occurred (32.5% vs. 21.3%, *p* = 0.348; 34.4% vs. 21.8%, *p* = 0.304). In addition, the 1‐year mortality rates were significantly higher with MDD (30.5% vs. 9.1%, *p* = 0.007; 20.0% vs. 4.3%, *p* = 0.036) and anxiety (31.4% vs. 11.1%, *p* = 0.010; 21.9% vs. 5.5%, *p* = 0.031) for all PDAC patients and those who received radical surgeries. Tables 5 and 6 summarize the associations and effects of different potential influencing factors on 1‐year mortality. Among them, MDD status (OR: 4.390, 95% CI: 1.501–12.843; OR: 5.625, 95% CI: 1.119–28.265) and anxiety status (OR: 3.657, 95% CI: 1.368–9.778; OR: 4.853, 95% CI: 1.157–20.365) were significantly correlated and contributed to a higher 1‐year mortality rate for both all patients and those that received radical surgeries.

**TABLE 5:** Univariate analysis of factors for 1‐year mortality in all PDAC patients.

Variables	OR	95% CI	*p*‐value
Age	1.029	0.978–1.081	0.268
Sex (male)	0.510	0.192–1.359	0.178
BMI	0.961	0.847–1.089	0.532
ASA (II + III)	1.456	0.573–3.702	0.430
With additional medical insurance	0.791	0.300–2.084	0.635
Income	0.808	0.446–1.463	0.481
With college education	2.118	0.705–6.365	0.181
Married	–	–	1.000
Working status (employed)	1.293	0.473–3.538	0.616
With self‐care ability	0.471	0.108–2.045	0.315
Tumor pain	1.330	0.532–3.326	0.542
Weight loss >3 kg	3.873	1.488–10.082	0.006
Diabetes	1.901	0.727–4.972	0.191
Hypertensions	0.853	0.328–2.221	0.745
Cardiac or cerebral diseases	0.825	0.216–3.152	0.778
Hb	0.986	0.959–1.014	0.336
TBil	1.001	0.995–1.006	0.836
ALB	0.899	0.820–0.986	0.024
CA199 > 37 U/mL	3.616	0.998–13.103	0.051
Smoke	1.654	0.564–4.852	0.359
Alcohol	1.053	0.346–3.201	0.928
Treatments (radical surgery)	0.140	0.051–0.381	< 0.001
T stage ≥ III	5.425	1.194–24.651	0.029
Metastasis	5.857	1.981–17.315	0.001
N stage (N_1_ or N_2_)	0.538	0.182–1.586	0.261
Hospital stay	1.017	0.979–1.056	0.382
With MDD	4.390	1.501–12.843	0.007
With anxiety	3.657	1.368–9.778	0.010
Physical disorder during chemotherapy	1.037	0.383–2.807	0.943
Mental disorder during chemotherapy	0.595	0.230–1.542	0.285
Anti‐therapy emotions	0.459	0.182–1.160	0.100
Chemotherapy suspension	2.925	1.146–7.465	0.025

Abbreviations: ALB, albumin; ASA, American Society of Anesthesiologists; BMI, body mass index; CA199, carbohydrate antigen199; CI, confidence interval; Hb, hemoglobin; MDD, major depressive disorder; OR, odds ratio; PDAC, pancreatic ductal adenocarcinoma; TBil, total bilirubin.

**TABLE 6:** Univariate analysis of factors for 1‐year mortality in patients receiving radical surgeries.

Variables	OR	95% CI	*p*‐value
Age	1.045	0.963–1.134	0.294
Sex (male)	0.488	0.117–2.029	0.324
BMI	1.068	0.911–1.252	0.418
ASA (II + III)	2.158	0.519–8.969	0.290
With additional medical insurance	1.500	0.294–7.645	0.626
Income	0.972	0.399–2.365	0.950
With college education	1.354	0.256–7.175	0.722
Married	–	–	1.000
Working status (employed)	1.221	0.288–5.182	0.786
With self‐care ability	–	–	0.999
Tumor pain	0.843	0.220–3.229	0.803
Weight loss >3 kg	2.850	0.746–10.886	0.126
Diabetes	3.053	0.797–11.699	0.104
Hypertensions	1.567	0.418–5.874	0.506
Cardiac or cerebral diseases	2.571	0.576–11.473	0.216
Hb	0.995	0.951–1.042	0.841
TBil	1.002	0.995–1.008	0.586
ALB	0.940	0.822–1.074	0.363
CA199 > 37 U/mL	1.260	0.301–5.272	0.752
Smoke	3.282	0.810–13.291	0.096
Alcohol	2.110	0.481–9.251	0.322
T stage ≥ III	5.745	0.692–47.676	0.105
N stage (N_1_ or N_2_)	1.484	0.396–5.558	0.558
Hospital stay	1.089	1.031–1.149	0.002
With MDD	5.625	1.119–28.265	0.036
With anxiety	4.853	1.157–20.365	0.031
Physical disorder during chemotherapy	0.552	0.109–2.798	0.473
Mental disorder during chemotherapy	0.285	0.057–1.428	0.127
Anti‐therapy emotions	0.381	0.099–1.466	0.160
Chemotherapy suspension	5.478	1.301–23.073	0.020
One‐year recurrence or metastasis	8.896	2.065–38.315	0.003

Abbreviations: ALB, albumin; ASA, American Society of Anesthesiologists; BMI, body mass index; CA199, carbohydrate antigen199; CI, confidence interval; Hb, hemoglobin; MDD, major depressive disorder; OR, odds ratio; PDAC, pancreatic ductal adenocarcinoma; TBil, total bilirubin.

## 4 DISCUSSION

In this study, we analyzed independent risk factors for MDD and anxiety in patients with PDAC. The non‐biological factor of employment status and patients with unresectable tumors were identified as independent risk factors for MDD and anxiety. After the occurrence of MDD and anxiety, there were significant decreases in several aspects of the patients' QoL. Furthermore, worse chemotherapy compliance was observed during follow‐up, and the potential association of MDD and anxiety with the 1‐year OS outcomes should also be highlighted. As a high proportion of patients in our cohort was diagnosed with MDD or anxiety, clinicians should attach great importance to the mental care.

Previous studies have shown that non‐biological factors can affect prognosis in many tumor types.^15^, ^19^ We included several potential factors that might influence mental status in our analysis and the employment status was proved to affect the occurrences. However, the mechanism behind this is unclear, and there were no similar study designs associating non‐biological factors with MDD or anxiety disorders in extant literatures as far as we know. From our perspective, psychological and sociological explanations may be crucial and we speculated that those PDAC patients still employed were more likely to develop MDD or anxiety because they might have more worries about plans and the balance between work and treatment. Returning to work is an important clinical outcome^20^ and is highly associated with mental health change^21^ in cancer survivors. Another important thing is that many other social factors were hard to be quantized and they indeed could affect the results. For instance, we did not contain patients' cognition in the non‐biological factors for lack of classification criteria, however it was proved to play a role in mental disorders.^22^, ^23^ In our cohort, though all patients were well‐informed of their disease, their awareness and acceptability toward PDAC was unclear. An overly clear understanding of diseases may not always be helpful, and patients may experience more worry, nervousness, and anxiety, especially when facing highly malignant tumors such as PDAC. On the contrary, family members' attitude and care also differ and a few families might conceal the true condition of tumor progression to give patients more comfort. Therefore, such invisible factors would definitely have an impact on the study results and should be taken into account in real situations. Furthermore, in our opinion, MDD and anxiety might occur due to the great mental attack when patients were informed of no chances for radical surgeries and potential limited lifetime.

In our cohort, we demonstrated that MDD was often accompanied by anxiety, leading to worse chemotherapy compliance and 1‐year survival result. In fact, MDD and anxiety may occur before the cancer diagnosis and already exist when the therapy begins, thus affecting the prognosis both pre‐ and postoperatively. Previous researches mainly focused on the impact of preoperative mental disorders before the cancer diagnosis and revealed that the incidences of a reduction in tumor resection and more short‐term surgical adverse events as well as less chemotherapy acceptance and palliative care were significantly higher and less ideal prognosis were foreseen in patients with MDD and anxiety.^4^, ^24^, ^25^ In our study, it was quite novel that MDD or anxiety were assessed after operations or biopsies, and our aim was to get a cancer‐related or therapy‐related result. Aside from MDD and anxiety, it is obvious in our result that the 1‐year OS could be influenced by tumor characteristics and the therapy. Moreover, we demonstrated that the chemotherapy suspension potentially determined the 1‐year OS both in all PDAC patients and the subgroup of patients undergoing radical surgeries. From our perspective, it might be that MDD and anxiety affected patients' physical or mental health and caused anti‐therapy emotions, thus the worse compliance led to poorer treatment outcomes.

Ultimately, MDD or anxiety can predict worse QoL, accompanied by emotional distress and symptoms. Early interventional therapies have been proven to treat MDD and anxiety efficiently, which could significantly improve QoL.^26^ Furthermore, the distressing QoL also interfere with the physical and mental health, affecting attitudes and compliances change during the chemotherapy. Therefore, having the ability to maintain a good QoL is also an inevitable consideration in clinical decision‐making and early identification as well as intervention may help improve survival outcomes.^27^ Potential mechanisms for accelerating tumor progression may be the neurobiological pathways including inflammation, stress, decreased immune surveillance, and autonomic and HPA axis functions.^2^

This study has several limitations. First, a selection bias existed because of the study design, and the questionnaire results mainly depended on the patients' subjective feelings. Second, the diagnosis of MDD and anxiety was based on a quick questionnaire using the PHQ‐9 and GAD‐7, and the incidence may vary significantly according to different diagnostic methods. Third, all data were obtained from a single center. Although the sample size was small, it should be acknowledged that few patients agreed to participate in the survey. This is the largest longitudinal study in the Chinese population to reveal the impacts of MDD and anxiety in several aspects. Further external validation with a larger sample size is required to confirm general applicability. Finally, other important clinical data, such as mental interventions and drug use, which could also contribute to both short‐ and long‐term differences, were absent.

## 5 CONCLUSIONS

Our study investigated the incidence and risk factors for MDD and anxiety in a Chinese population and confirmed their adverse influences on QoL, chemotherapy compliance, and survival outcomes. Therefore, mental health assessments and appropriate interventions should be applied in treating patients with PDAC.

## AUTHOR CONTRIBUTIONS

**Yuchen Ji:** Data curation (equal); methodology (equal); writing – original draft (equal); writing – review and editing (equal). **Haoda Chen:** Data curation (equal); formal analysis (equal); methodology (equal); writing – review and editing (equal). **Yuxuan Yang:** Data curation (equal); formal analysis (equal); writing – review and editing (equal). **Yiran Zhou:** Methodology (equal); supervision (equal); validation (equal); writing – review and editing (equal). **Hongzhe Li:** Conceptualization (equal); investigation (equal); resources (equal); writing – review and editing (equal). **Haiyan Jin:** Data curation (equal); funding acquisition (equal); project administration (equal); resources (equal); supervision (equal); validation (equal); writing – review and editing (equal). **Junjie Xie:** Conceptualization (equal); funding acquisition (equal); project administration (equal); resources (equal); supervision (equal); validation (equal); writing – review and editing (equal). **Baiyong Shen:** Conceptualization (equal); funding acquisition (equal); project administration (equal); resources (equal); supervision (equal); writing – review and editing (equal).

## CONFLICT OF INTEREST STATEMENT

The authors have no conflict of interest to declare.

## ETHICAL APPROVAL

This study conformed to the provisions of the Declaration of Helsinki (as revised in 2013). All the patients included signed informed consent forms and agreed to data collection. It was approved by the institutional review board of our hospital.

## Data Availability

The data supporting the findings of this study are available from the corresponding authors upon reasonable request.
